# A Digital Assistive System for Maintaining Nutrition and Mobility in Older Adults: Usability and Feasibility Findings From a Pilot Study

**DOI:** 10.2196/89681

**Published:** 2026-05-13

**Authors:** Mareike Foerster, Marianne Timper, René Puhlemann, Vincent Quinten, Rebecca Diekmann

**Affiliations:** 1 Department of Health Services Research, School of Medicine and Health Sciences, Carl von Ossietzky Universität Oldenburg, Ammerländer Heerstraße 114-118, Oldenburg, Lower Saxony, 26129, Germany, +49 4417984882; 2 Assistive Technologies for Care and Health Professionals Group, OFFIS e.V. - Institut für Informatik, R&D Division Health, Oldenburg, Germany

**Keywords:** technical assistance system, gerontechnology, interactive health promotion, older adults, independence, nutrition, mobility, usability, feasibility

## Abstract

**Background:**

Due to demographic changes, the number of older people is increasing, often accompanied by limitations in mobility, nutrition, and independence. Preventive monitoring is rare, as care systems struggle with staff shortages and limited resources. Technical assistance systems can support older people in self-assessing their health and maintaining independence. We developed the AS-Tra system, which combines an application with a measurement and training station (MuTS), to enable early detection of nutrition and mobility-related deficits and risks.

**Objective:**

This paper presents the pilot study of the AS-Tra system with the aim of evaluating its usability and testing the feasibility of collecting health-related data from older adults (≥70 y) with early/mild deficiencies in nutritional state and mobility in preparation for a future randomized controlled trial.

**Methods:**

The system used in this 4-week pilot study was developed as a complex intervention in accordance with the Medical Research Council framework. Participants (target n=10) were recruited through a participant registry. They completed standardized mobility assessments (grip strength, Timed “Up and Go,” and 5-Time Chair Rise) at baseline and after 1, 2, and 4 weeks (T0, T1, and T2, respectively). Mini Nutritional Assessment—Short Form and short physical performance battery were recorded at baseline and at T2. Participants received a tablet app for regularly documenting nutrition and an activity sensor for 7 days of physical activity monitoring and performed weekly training starting at T0. At T2, the System Usability Scale (SUS) and feedback questionnaires (Evaluation Overall System [EOS] questionnaire—the evaluation of all subcomponents on a scale of 1-5, weekly Experience Report) were additionally collected. Data were analyzed descriptively using IBM SPSS Statistics, in which data were shown as total numbers, percentages, and means with SDs, and data from the activity sensor were displayed and analyzed using Python.

**Results:**

A total of 9 older adults, with 1 dropout (mean 80, SD 5 y, 50% female), participated in this study. The SUS score was good (mean 79, SD 13.4 points). The MuTS devices had minor technical problems (in <17% of MuTS sessions), while 57% (17/30) of the users experienced instability issues with the food diary in the tablet app. The average overall system ratings were positive, with an EOS score of 2.01 (SD 0.99).

**Conclusions:**

The usability of the technical assistance system used in this study was rated as good. The data collection using questionnaires, sensors, and automated assessments proved feasible. The biggest challenge was the tablet-based food diary, which still needs improvement before the effectiveness of the AS-Tra system regarding mobility and nutritional status can be evaluated in a randomized controlled trial.

## Introduction

As a result of demographic changes and increasing life expectancy, the proportion of older adults within the population is increasing significantly relative to other age groups [1]. This demographic shift is accompanied by a number of age-related challenges, including a decline in cognitive function, physical performance, and sensory perception [2]. Decreased muscle strength is also common, highlighting the importance of good nutritional status and regular physical activity in older age [3]. Physical activity and a balanced diet are key factors in promoting healthy aging and are crucial for maintaining or enhancing functional independence and reducing the risk of requiring long-term care [3,4].

Despite their importance, routine monitoring of mobility and nutritional status is often limited in daily care practice. This is frequently attributed to a lack of time and insufficient awareness or training among health care providers [5]. In addition, the current and projected shortage of caregivers poses a further challenge to the sustainability of traditional care models in meeting the needs of an expanding older population [6]. Physiotherapy, nutritional therapy, or other therapies initiated after acute illness or hospital discharge are often discontinued upon return to the home environment, and sustained behavioral change is rarely observed [7].

Technological interventions—particularly in the form of technical assistance systems—have been proposed as a possible supplement to conventional care, as they enable older adults to perform health-related assessments and activities independently in their home environment. Such systems can facilitate the ongoing monitoring of nutrition and mobility with much greater regularity than traditional forms of care, such as primary care. Moreover, they can contribute to the early detection of health risks and the prevention of adverse outcomes [8]. As part of more comprehensive, complex interventions, these systems could also help to close knowledge or behavioral gaps related to mobility and nutrition.

In general, technical assistance systems comprise digital technologies that aim to promote autonomy and quality of life in older age. When these systems are used in the home environment, they can support the health process through functions such as measuring vital parameters or encouraging physical activity. Often, their main goal is the early detection of changes in health status and the promotion of preventive strategies [9]. Most currently available technologies for older adults are mobile eHealth applications for smartphones or tablets, which can typically be used for medication adherence [10,11], self-monitoring of dietary intake [12,13], or tracking physical activity via fitness devices [14-16].

However, comprehensive approaches that simultaneously cover several health areas, such as mobility and nutrition, through integrated technical solutions are still limited. While applications such as NuMobe and KOKU-Nut [17,18] attempt to address both areas, they do so without incorporating broader technical infrastructures, such as measurement or training stations. In principle, more holistic systems could provide a more complete representation of an individual’s health status and thereby support a more effective aging process [19].

Nevertheless, the adoption of such technologies by older adults is not without challenges. The difficulties in accessing and operating digital devices, whether at self-service checkouts in retail or when using touch screen-based applications, are well-documented [20]. Therefore, the design and implementation of technical assistance systems must take a user-centered approach that takes into account the specific skills, (dis)abilities, and limitations of older adults with generally lower technical readiness [20].

In this paper, we describe the results of a pilot study to test the usability of the technical assistance system AS-Tra (Assistance System for sustainable improvement of nutritional and mobility status of older adults [≥70 y] under consideration of the transtheoretical model of health behavior change [TTM]). The system was developed using a user-centered approach and builds on the results of previous studies conducted at our university [17,21-25].

The primary objective of this study was to test the usability of the overall system, consisting of the measurement and training station (MuTS) and the tablet-based app, evaluated with older adults (≥70 y) with increased risk of or early or mild nutritional and movement deficits over a 4-week period. Evaluating health effects was not part of this study. Additionally, the study aimed to assess the feasibility of collecting health-related data on nutritional status, strength, and physical activity to capture behavioral change in preparation for a subsequent randomized controlled trial (RCT).

## Methods

### Technical Assistance System

#### Overview

The AS-Tra system was developed based on the phases of the Medical Research Council framework for complex interventions. The context analysis included focus group discussions to identify user preferences and perceived barriers to technology adoption [21,26]. Furthermore, it identified the evidence base based on the current literature and inputs from experts in the field. The findings from these discussions were incorporated into the system design, which were then refined through a user-centered design process involving iterative usability testing [27]. The system was developed with the conceptual aim of enabling users to carry out exercises and assessments independently in a central location while continuing to engage with the tablet app in their everyday lives. To promote long-term behavioral change in the areas of nutrition and mobility, the app’s content was tailored to the TTM [28,29]. This concept for describing, explaining, and influencing health-related behavioral changes comprises 6 stages, from the precontemplation phase (no intention to change behavior), through preparation (planning the behavior change), to maintenance or relapse [29]. For the design of the app, it implies that new content should be unlocked in the app, which the user was previously unable to see, when higher stages in the TTM are reached. This is intended to motivate users to continuously work on their nutrition and exercise status while preventing them from feeling overwhelmed. In this study, we evaluated the feasibility of data collection to determine the TTM-stage as objectively as possible.

The AS-Tra system consists of 2 core components: a MuTS, which enables objective and automated measurements and exercises of physical function, and a tablet-based app. The system integrates multiple functional assessments described in detail.

#### Measurement and Training Station

The following measurement and training modules were integrated into the AS-Tra system: pulse measurement, hand grip strength, Timed “Up and Go” (TUG), 5-Time Chair Rise Test (5-CRT), and interactive exercises. Pulse is measured with an optical sensor (Polar OH1). Hand grip strength, an indicator of loss of function [30], was measured using the K-Force Grip (SAS Kinvent Biomechanique), which is connected to the MuTS via Bluetooth.

Mobility was assessed using the TUG test, which evaluates the risk of falling and general functional mobility [31,32]. For this purpose, we used an automatic Timed “Up and Go” (aTUG) chair equipped with infrared sensors that automatically recognize the seat occupancy and measure the test duration [23,24]. The aTUG chair was also used for the 5-CRT, which records the time needed to stand up from the chair and sit down 5 times as quickly as possible.

Exercises were facilitated by the THERA-Trainer Senso (model Senso 642), which has 5 force plates to track movement. The accompanying Dividat Play (Dividat AG) software offers gamified exercise programs focusing on coordination, balance, and endurance.

**Figure 1.**
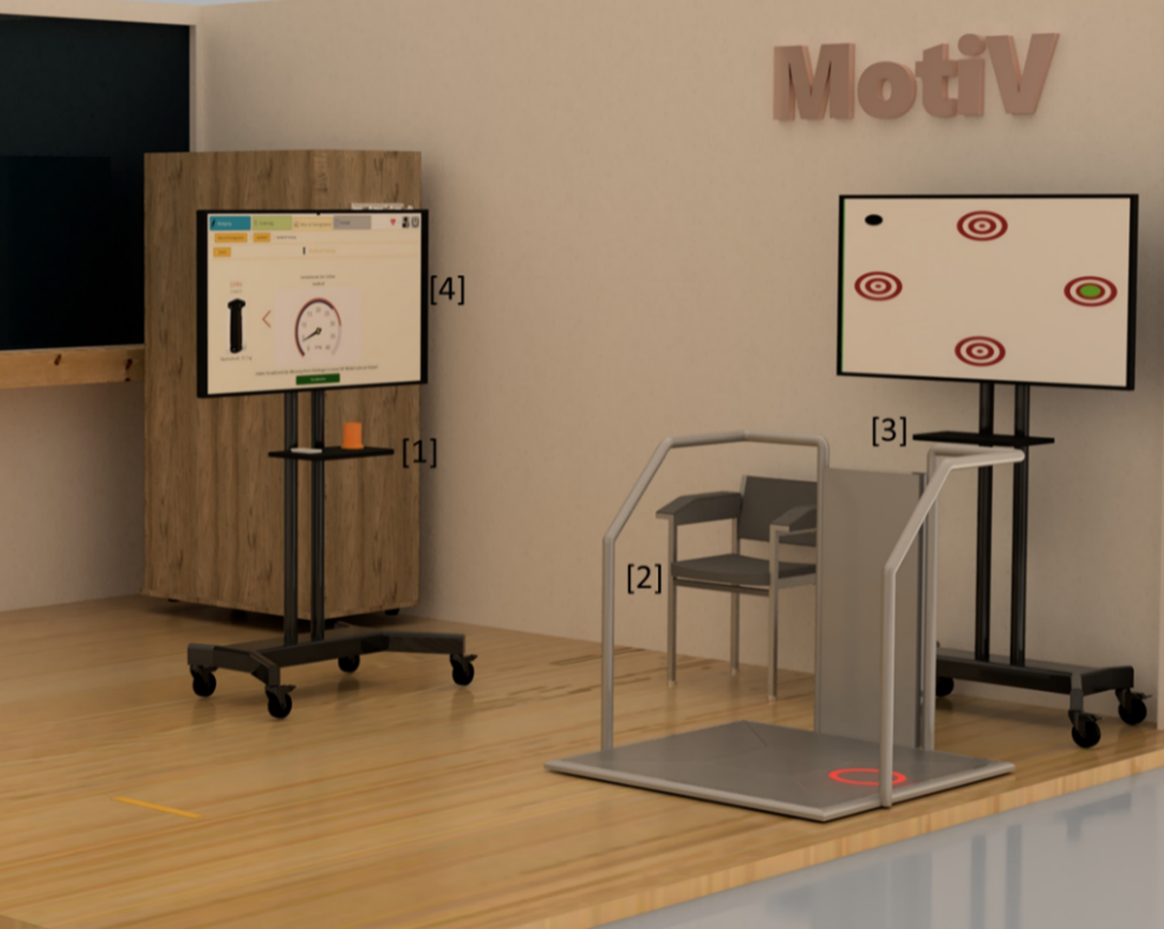
Schematic representation of the measurement and training station of the AS-Tra project featuring a table on which the Polar OH1 pulse measurement device and the K-Force Grip Kinvent hand grip measurement device are placed [1], the aTUG chair [2], the THERA-Trainer Senso training device [3], and the 55-inch touch screen with the app [4]. AS-Tra: Assistance System for sustainable improvement of nutritional and mobility status of older people (≥ 70 y) under consideration of the transtheoretical model of health behavior change; aTUG: automatic Timed “Up and Go” test.

A schematic representation of the MuTS and its integrated devices is presented in Figure 1.

#### Application

The present application was further developed on the basis of the already evaluated NuMob-e app, which represents an age-appropriate electronic coaching system designed to support older adults with nutritional and physical activity deficits, particularly for geriatric rehabilitation patients [17,21,22]. To improve device compatibility, the app was migrated from the Android platform to the Microsoft .NET multiplatform app user interface framework, enabling native operation on both Windows and Android devices with a single code base. Additional features were implemented to integrate the application with the MuTS and associated assessment modules. Furthermore, the navigation was redesigned based on feedback from previous user tests.

The app interface has four main sections: (1) mobility, (2) nutrition, (3) measurement and training station, and (4) contact. The mobility and nutrition modules contain educational content, including informational texts, videos, and quizzes on topics such as physical activity recommendations, age-associated recommendations for healthy nutrition, and common misconceptions about nutrition [17,21,22]. Interactive features such as exercise and food diaries allow users to track their physical activity and food intake. Automated feedback is provided on indicators such as protein intake, fluid intake, and the duration and intensity of activity. The app also includes the Mini Nutritional Assessment—Short Form (MNA-SF) for screening the risk of malnutrition, which users can complete independently [33].

The Android app on the tablet is intended for everyday use at home. Participants took a tablet provided by the study team home with them. In the MuTS, the Windows version runs on a desktop computer connected to a 55-inch touch screen display and facilitates user interaction and connects to all MuTS devices (Figure 1, [4]). Each assessment or exercise module is started via corresponding buttons in the app on the screen and is accompanied by step-by-step text instructions and explanatory videos. Upon completion, the results are displayed both graphically and in written form, allowing users to compare and interpret their latest results, providing a visual overview of progress. This allows potential deterioration to be identified at an early stage and positive developments to serve as motivation [34]. Since all integrated devices are equipped with sensors and automatic connection via Bluetooth or through a server using a local area network connection, the recorded data are transmitted in real time and displayed as results for the user in the app on the screen. The exercise data are stored in a MySQL database on a server at the University of Oldenburg. This server can also be used to synchronize the tablet with the MuTS via Wi-Fi, so that all data can be viewed both in the tablet app and on the screen in the MuTS.

### Usability and Feasibility

Usability, defined as the extent to which users can achieve specific goals effectively and satisfactorily [35], was assessed using the System Usability Scale (SUS) developed by Brooke et al [36]. This standardized questionnaire contains 10 statements about the usability of a system. A 5-point Likert scale is used to answer the questions. For the classification of the SUS score, we used the adjective rating scale of Bangor et al [37]. A total of 100 points can be achieved, which can be divided into the following categories: best (100.0‐84.1 points), excellent (84.0‐80.8 points), good (80.7‐71.1 points), okay (71.0‐51.7 points), poor (51.6‐25.1 points), and worst possible (25.0‐0.0 points) [36,37].

In addition, technological commitment, according to Neyer et al [38], was recorded to reflect technical readiness, which encompasses technical acceptance, competence, and control. A 5-point scale with the response options strongly disagree (1), disagree (2), somewhat disagree (3), somewhat agree (4), and strongly agree (5) is used to assess attitudes toward and use of modern technology in general [38].

Regarding the feasibility of using the entire assistance system, this study focuses on the participants’ experiences, data collection, storage, processing, and analysis. To identify the strengths and weaknesses of the MuTS and the tablet app, participants complete a report after each use of the assistance system documenting the use of each individual integrated module (see Figure 1). Finally, a self-developed questionnaire was used to evaluate the overall system (see Multimedia Appendix 1). This questionnaire consists of 10 questions, which can be answered on a scale of 1 to 5, with 1 being “very positive” and 5 being “very negative.” The questions relate to the content and use of the tablet app, the handling of the equipment in the MuTS, the exercise, and the general process. In addition, 5 open-ended questions were included to gather feedback on the content that needs improvement, problems in the process, what participants liked best, and the extent to which they believe that the MuTS, including app, can influence their nutritional and mobility behavior. The participants’ experiences with the overall system will inform the testing and trial of the MuTS, the app (tablet-based and in the MuTS), and data collection for a planned subsequent RCT. Furthermore, analysis algorithms will be developed to evaluate the data, facilitating the RCT.

### Nutritional and Mobility Data

The German version of the MNA-SF (0‐14 points) was used to screen for malnutrition and risk factors. It contains 6 questions to differentiate between normal nutritional status (12‐14 points), risk of malnutrition (8‐11 points), and malnutrition (0‐7 points) [33,39]. In order to gain better insights into dietary habits, participants were asked to maintain a food diary in the tablet app for 3 consecutive days during the week preceding T0, T1, and T2. The data collected were screened by study staff members for calories, protein, and fat intake. In addition, the fluid and food intake were evaluated according to the recommendations of the German Nutrition Society, specifically for older adults [40].

The short physical performance battery (SPPB) and the TUG test were used to assess possible mobility deficits and to track their development over time, as the tests were performed at baseline, T0, T1, and T2. The SPPB consists of 3 parts to assess physical functions: static balance, walking speed, and the time it takes to get up from a chair 5 times (5-CRT). The rating scale contains 3 categories: poor (0‐4 points), moderate (5‐8 points), and good mobility (9‐12 points) [41,42]. The TUG is a test to measure the time it takes a person to get up from a chair (if possible, without the help of the arms), walk 3 meters, turn around, walk back, and sit down on the chair again. Walking speeds above 0.8 m/s were considered normal [23,43].

### Activity Sensor

In addition to recording the health status using the questionnaires mentioned above, an activity sensor (Axivity AX6) was used to objectify physical activities. Therefore, the sensor was attached to the front of the participants’ thighs for 7 days, a week before T0, T1, and T2. The sensor has 6 axes and records linear acceleration and angular velocities [44]. This allowed movement data to be recorded over 7 days and analyzed according to the World Health Organization (WHO) global guidelines on physical activities [45]. Using a cut point analysis from Skjødt, the sensor data were analyzed for minutes of movement and their intensity [46]. This metric objectifies the participant’s weekly exercise duration, categorizing sedentary, light, moderate, and intense activity.

### Participants and Recruitment

Potential participants aged 70 years and over were recruited via our register of people who had previously expressed interest in participating in the AS-Tra project. Other recruitment strategies included the distribution of flyers in places frequently visited by older adults, such as physiotherapy practices, senior centers, and care support centers, as well as public relations work through press releases and newspaper articles. Interested individuals were requested to contact the study team by telephone or email. Subsequently, a consent form to be included in the registry, along with the MNA-SF and the inclusion and exclusion criteria, was sent by mail to those who expressed interest. Based on the initial data collected, it was possible to assess whether the individual is suitable for the study. This information was also recorded in the registry, which automatically created an order in which the subjects are requested for the study. If the individuals contacted were not interested, they were asked whether they no longer wished to be included in the directory or whether they may be contacted again at a later date for possible follow-up studies. Recruitment began one and a half weeks before the start of the study and continued throughout the study until 10 participants were included. The age group above 70 years was selected because it differs significantly from the mid-60s age group, many of whom are still working [47]. The risk of health impairments continues to increase in the over-70 population [48], and digital support, which plays an important role in prevention, is often limited or entirely absent [49,50]. In addition, several components from previous projects, including the app, were already tailored to older, fit users (aged 70 y and above) or specifically designed for geriatric and rehabilitation patients (eg, the aTUG chair) [17,21-24].

In this study, eligible participants were those who met at least one of the following inclusion criteria:

1. risk for malnutrition or malnutrition as defined by the MNA-SF (<12 points) [33]
2. presence of one or more of the following risk factors for malnutrition:
  - recent unintentional weight loss (within the past 3 mo)
  - reduced food intake during the same period
  - low BMI <22 kg/m²
3. impaired mobility, defined by a walking speed of <0.8 m/s
4. a score of ≤8 points on the SPPB [41].

Exclusion criteria included (1) inability to provide informed consent, (2) physical inability to visit the study center, (3) inadequate understanding of the study process or the German language, (4) severe mobility impairments that prevent locomotion, and (5) severe visual impairments that preclude the independent use of the system.

All participants were informed about the study by a member of the study team. The procedure was described in detail and questions could be asked.

### Study Design and Procedure

The pilot study was carried out from October 2024 to February 2025 in accordance with the study protocol approved by the medical ethical committee of the Carl von Ossietzky University Oldenburg, Germany (2024‐116). The AS-Tra system was evaluated over 4 weeks per participant. Participants used the MuTS on their own, not simultaneously with other participants.

**Figure 2.**
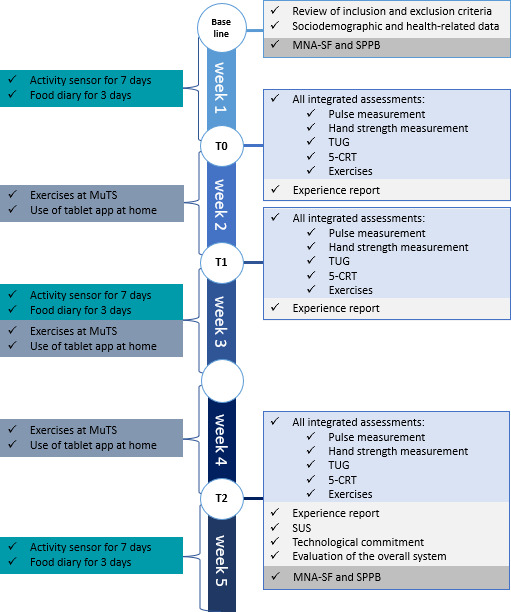
Flowchart of the pilot study’s procedures over 5 weeks for each participant with the 4 measurement points from baseline to T2 and a list of measurements and data collection using questionnaires on the right side and the times of the exercises, food diary entries, and the wearing of the activity sensor on the left side. 5-CRT: 5-Time Chair Rise test; MNA-SF: Mini Nutritional Assessment—Short Form; MuTS: measurement and training station; SPPB: short physical performance battery; SUS: System Usability Scale; TUG: Timed “Up and Go” test.

Figure 2 shows a schematic overview of the study’s procedures, highlighting the 4 measurement points baseline (wk 0), T0 (wk 1), T1 (wk 2), and T2 (wk 4), as well as the corresponding assessments, questionnaires, and data collection periods (digital food diary activity sensor).

All appointments (baseline-T2) were accompanied by at least one trained study staff member to observe the use of the system and to record the observations in the form of an interview format using questionnaires.

At baseline, after checking the inclusion and exclusion criteria, sociodemographic and health-related data, the MNA-SF, and the SPPB were recorded, and the participants received a tablet with the app to use in their home environment. Furthermore, an Axivity sensor was attached to the front of the participant’s thigh.

At T0 (after 1 wk), the participants returned to the MuTS, and the use of all elements in the MuTS (see Figure 1) was explained. All assessments (listed in Figure 2) were carried out together with the study staff. For the following 3 weeks, participants were encouraged to use the MuTS independently for regular assessments and to test the tablet app at home. They were recommended to do exercises twice a week on the THERA-Trainer Senso.

At T1 (after 2 wk), all assessments were carried out again. At the last appointment (T2), after 4 weeks, the final measurement took place, including all assessments. In addition, questionnaires were collected to evaluate the SUS, technological commitment, and the Evaluation Overall System (EOS) questionnaire (see Multimedia Appendix 1). Moreover, the MNA-SF and the SPPB were repeated. In week 5, participants wore the activity sensor and used the digital food diary for 3 consecutive days, but they were no longer allowed to use the MuTS.

At T0, T1, and T2, participants completed the Experience Report (ER) questionnaire (see Multimedia Appendix 1), documenting the use of the MuTS and the tablet app.

As shown in Figure 2, the activity sensor was worn for 7 days in week 1, week 3, and week 5. For each of those weeks, participants were required to maintain a food diary for 3 consecutive days. This allowed the collection of nutritional and activity data from each participant at 3 points during the study.

### Data Collection

All collected data, including sociodemographic information, medical history, MNA-SF, SPPB, and technology-related data such as technology commitment, SUS, ER, and EOS, were collected using a combination of paper and digital methods. For older adults, questionnaires were provided on paper if preferred, while most data were collected digitally through the REDCap (Research Electronic Data Capture; version 14.5.44) data management system by the study staff members [51,52]. The paper-based records were digitized by the study staff using a dual entry method.

### Data Analysis

Participants’ characteristics, the data of the MNA-SF, the SPPB, and the TUG were analyzed descriptively using SPSS (version 29.0.0). The data were presented as total numbers, percentages, and means with SDs or medians with IQRs depending on the distribution. The data of the ER and the EOS questionnaires were also evaluated descriptively and presented as total numbers and percentages.

The mean values, including SDs of the MNA-SF and SPPB scores of all participants, were graphically processed and color-coded to indicate the scores achieved in both questionnaires at the baseline and end of the study.

Data from the activity sensor were analyzed using the procedure described above and displayed graphically using a Python script. The number of minutes of moderate activity during the weeks the sensor was worn was visualized to show its progression over the 4-week study period. In addition, these values were compared to the recommendation of the WHO guideline value of 150 minutes of moderate activity per week [45].

### Ethical Considerations

The study was approved by the Ethical Review Board of the Carl von Ossietzky Universität Oldenburg (2024‐116). All participants were informed of the aim of the research, the methods used, how the results would be presented, and the right to decline to participate in the study or to withdraw consent to participate at any time without further explanation. All participants provided written informed consent before data collection. All data were stored pseudonymously, which means that without a key list, it is not possible to link personal data to research data. Only study staff have access to the key list. All study participants received an expense allowance of €30 (US $35.28). If they did not participate in the study until the end, they received €5 (US $5.88) instead.

## Results

### Characteristics of the Included Participants

A total of 10 older adults (5 females and 5 males) were included in the pilot study. One participant dropped out after T0, after 1 week of participation due to illness, so that data from all 10 participants at baseline and 9 at T2 were included in the final analysis.

In total, there are complete datasets from 6 participants, that is, excluding the 67% dropout rate. For the remaining 3 participants, datasets from the food diary are unavailable. In one case, the diary was not filled out at all, and in the other 2 cases, it was only filled out half as often as desired. Otherwise, all data are available. In addition, all participants adhered to the MuTS usage recommendations, were present at least once a week, regularly performed the assessments, exercised, and wore the activity sensor, as shown in Figure 2.

The average age was 80 (SD 5) years, with an average BMI of 23.7 (SD 5) kg/m² among female participants and 25.0 (SD 6.3) kg/m² among male participants. The baseline characteristics and T2 data of MNA-SF and SPPB, as well as the SUS and technology commitment score, are shown in Table 1. The SUS score at T2 was good (mean 79.3, SD 13.4 points). The mean score of technology commitment, assessed at T2, was 42.2 (SD 7.6) points.

**Table 1.** Characteristics of the participants (N=10, with 1 dropout after T0) and the results of the Axivity data (at baseline, T1, and T2) of the questionnaires MNA-SF, SPPB (at baseline and T2), SUS, and TC score (at T2).

Characteristics and variables	Values
Gender, n	
Male	5
Female	5
Age (y), mean (SD)	80 (5)
Walking aids, n (%)	5 (50)
Axivity data (min), mean (SD)	
Total minutes measured	
Baseline (N=10)	9891.2 (455.8)
T1 (n=9^a^)	9874.0 (438.0)
T2 (n=9^a^)	10,024.4 (106.5)
Moderate activity	
Baseline (N=10)	358.5 (226.6)
T1 (n=9^a^)	353.5 (216.4)
T2 (n=9^a^)	318.8 (162.5)
Number of people ≥150-min moderate activity, n (%)	
Baseline (N=10)	8 (80.0)
T1 (n=9^a^)	8 (88.9)
T2 (n=9^a^)	8 (88.9)
MNA-SF^b^, n (%)	
Normal	
Baseline (N=10)	5 (50)
T2 (n=9^a^)	7 (78)
Risk	
Baseline (N=10)	3 (30)
T2 (n=9^a^)	2 (22)
Malnutrition	
Baseline (N=10)	2 (20)
T2 (n=9^a^)	0 (0)
SPPB^c^**,** points (%)	
9-12	
Baseline (N=10)	2 (20)
T2 (n=9^a^)	4 (44)
5-8	
Baseline (N=10)	8 (80)
T2 (n=9^a^)	5 (56)
0-4	
Baseline (N=10)	0 (0)
T2 (n=9^a^)	0 (0)
Questionnaire score, mean (SD)	
TC^d^	42.2 (7.6)
SUS^e^	79.3 (13.4)

a One dropout after T0.

b MNA-SF: Mini Nutritional Assessment—Short Form.

c SPPB: short physical performance battery.

d TC: technology commitment.

e SUS: System Usability Scale.

### Nutritional and Mobility Data

**Figure 3.**
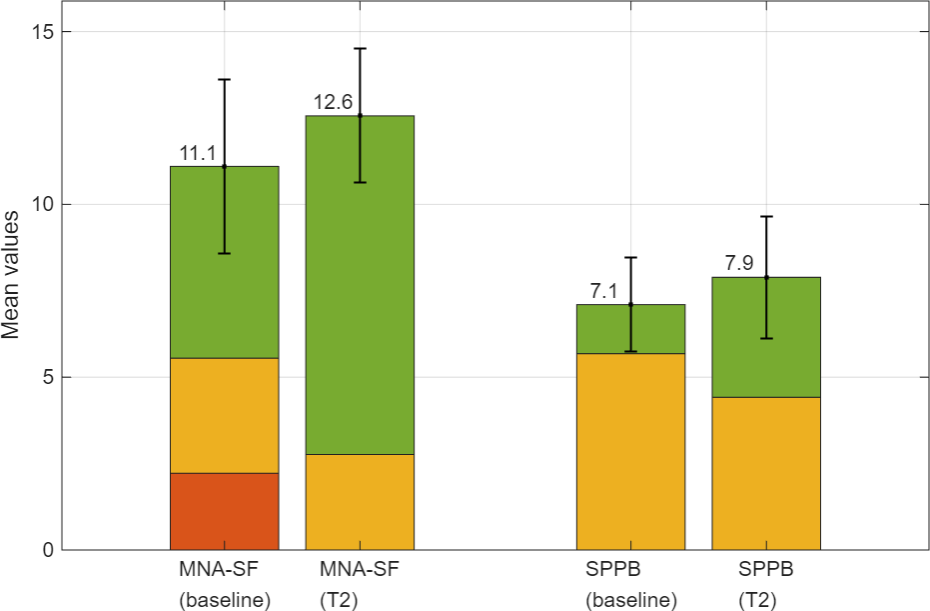
Mean and SDs of the MNA-SF (on the left) and the SPPB (on the right) at measurement points at baseline and T2 of the 4-week pilot study. The red color indicates the number of participants with malnutrition (MNA-SF) and poor mobility (SPPB); the yellow color indicates the number of participants at a risk of malnutrition (MNA-SF) and moderate mobility (SPPB); the green color indicates the number of participants with normal nutrition (MNA-SF) and good mobility (SPPB). Baseline includes data from 10 participants, whereas T2 only includes data from 9 participants due to 1 participant dropping out after baseline. MNA-SF: Mini Nutritional Assessment—Short Form; SPPB: short physical performance battery.

The results of the MNA-SF and SPPB recorded at times baseline and T2 of all participants are shown in Figure 3 as mean values and SDs. The color-code indicates whether malnutrition, a risk of malnutrition, or a normal nutritional status were present in the MNA-SF and whether poor mobility (0‐4 points), moderate mobility (5‐8 points), or good mobility (9‐12 points) were achieved in the SPPB. Descriptive changes in the results of these 2 questionnaires are visible at a glance.

**Figure 4.**
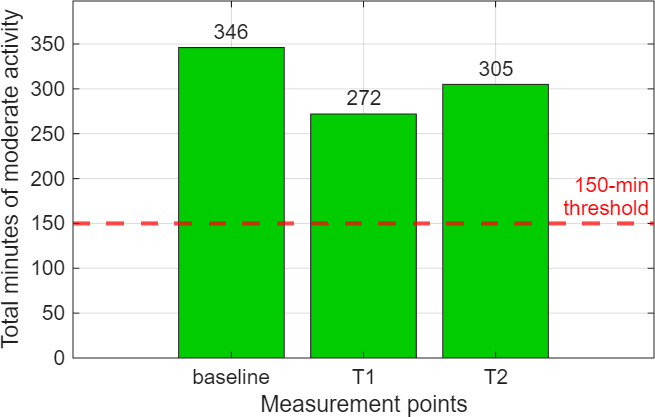
Exemplary number of minutes of moderate activity, measured with the Axivity sensor AX6 placed in the middle of the front of the thigh at the measurement points baseline, T1, and T2 of one participant. The red line shows the threshold of 150-minute moderate activity per week recommended by the WHO guideline. This participant reached the threshold at all 3 measurement points. WHO: World Health Organization.

Data from the activity sensor showed the total number of minutes recorded during the week in which the sensor was worn, including the number of minutes spent sitting or lying down. The recording duration of the Axivity sensor demonstrated high data completeness at all times. Moreover, the number of minutes of low, moderate, and high activities during that week at the time points baseline, T1, and T2 were measured in which the time spent on moderate activity is of particular interest in this study (see Table 1). Therefore, the graph (Figure 4) showing the number of minutes of moderate activity performed by a participant at the specified time points also includes the threshold value of 150 minutes per week recommended by the WHO guideline [45]. This threshold is represented by the horizontal red line for reference, making it possible to determine whether this value was achieved in one or more measurements or not. Overall, 7 participants exceeded the 150-minute threshold value in all 3 Axivity measurements during the 4-week study. In 1 out of 9 cases, this threshold value was exceeded in 2 out of 3 measurements, and 1 participant exceeded the 150 minutes once. Only 1 participant remained below the limit in every Axivity measurement.

### Usability and Feasibility

The SUS score was good (mean 79.3, SD 13.4 points). According to the ER of the participants and their EOS (see Multimedia Appendix 1), only minor problems were encountered during the use of the MuTS. Some technical problems occurred, particularly regarding the connection between devices in the MuTS, force plates that did not calibrate correctly automatically, the training plan that did not display correctly, or occasionally the touch screen that did not respond. All of these problems occurred at maximum in 17% of MuTS sessions.

The food diary was to be maintained 3 times per participant during the 4-week study. This resulted in a total of 30 uses for 10 participants. Due to 1 dropout, there was a total of 28 uses. In 22 of the 28 cases, entering data into the food diary led to problems and errors, and in 17 cases, it resulted in crashes, which corresponds to 57% (17/30) of the cases in which users attempted to enter nutritional data. Only in 4 of the 28 cases did no problems occur. This caused significant frustration for users, who were often unable to make further entries until they restarted the app.

The rating of the EOS with regard to individual points, such as content, handling, training program, process, and use, was generally between 1 and 2 on a scale of 1 to 5, where 1 corresponds to “positive” and 5 to “negative.” With regard to the tablet app and regular visits and use, the answers tended to be between 2 and 3. All exact mean values including SDs for the individual questions, calculated by scoring all 9 participants, can be seen in Table 2. Averaged across all questions of the EOS, the system was rated 2.01 (SD 0.99).

**Table 2.** EOS^a^ questionnaire to evaluate the overall system using a scale of 1 to 5, with mean values and SDs, formed by the responses of all 9 participants at T2.

Question	Classification on scale	Mean (SD)
The contents of the program are...	(1) Interesting to (5) uninteresting	1.4 (0.5)
Handling the equipment in the measuring and training station was...	(1) Easy to (5) difficult	1.8 (0.8)
The exercises on the Senso were…	(1) Easy to (5) difficult	1.9 (0.9)
The visit to the measuring and training station was...	(1) Structured to (5) chaotic	1.3 (0.5)
Making my own entries in the diet and exercise diary was...	(1) Good to (5) bad	2.1 (1.5)
The information from the app is useful for my everyday life...	(1) Useful to (5) useless	2.7 (1.2)
Using the tablet app regularly in my everyday life is...	(1) Easy to (5) difficult	2.8 (1.1)
Visiting the measuring and training station regularly in my everyday life is...	(1) Easy to (5) difficult	2.6 (1.2)
Finding specific content in the app when I’m looking for it is...	(1) Easy to (5) difficult	2 (1.3)
I like using the overall system...	(1) Good to (5) bad	1.6 (0.7)

a EOS: Evaluation Overall System.

Overall, the results show good usability (SUS, mean 79.3, SD 13.4) and also good feasibility, as only one of the 10 participants dropped out and the remaining 9 provided complete datasets. All participants adhered to the usage recommendations during the study. The only problems were with the tablet-based food diary, which crashed in 57% of cases, resulting in incomplete dietary data for 3 participants. This is also reflected in the EOS (see Table 2).

## Discussion

### Usability Evaluation

The aim of this study was to evaluate the usability for older adults (≥70 y), which can be classified as good (SUS, mean 79.3, SD 13.4), and to test the feasibility of data collection in preparation for a future RCT study, which was successful. Moreover, feedback on the use of the entire system was positive (EOS mean 2.01, SD 0.99; Table 2). The Axivity AX6 is a reliable wearable device for objectively recording activity data and classifying it into different intensity levels. Nutritional data were mainly recorded using the MNA-SF and the food diary in the tablet app, with the technical component of the tablet app proving to be the biggest problem in this study. This led to many crashes and instabilities in the app. This must be fixed before the RCT, as otherwise, there is little added value from the diary entries if the technical implementation causes several problems.

All of these main findings are discussed in more detail further, classified with the help of other studies, and interpreted.

The requirements of older adults for support services vary greatly and range from web-based solutions to personal counseling and exercise services [53]. Studies show that personal interaction and contact, for example, when using exergames, play a central role in the well-being of older people [54]. This suggests that independent exercises or measurements, where participants used the system alone, may have a more negative effect on usability compared to measurements carried out together with the study team. Previous studies with older adults showed similar or slightly higher usability scores for mostly app-based systems and, on average, younger participants [55-57]. All referenced studies included older adults aged 65 years and above, whereas in our study, the minimum age was 70 years. The average age in other studies was around the mid-70s, which is approximately 5 years below the average age in our study [55-57]. Their systems mainly focused on a specific health area [55-57]. In contrast, the AS-Tra system is a multidisciplinary, complex intervention that covers both mobility and nutrition and also includes the independent use of measuring and training devices. Due to this breadth of content and functionality, demands on the system design increase to ensure ease of use.

### Feasibility Evaluation

With regard to the feasibility of data collection, which was one of the objectives of this study, the questionnaires used proved to be meaningful and suitable for assessing the health status of older adults. The SPPB is a good predictor of physical performance and mobility in older adults, as it measures balance, walking speed, and lower extremity strength [42]. In addition, the participants in this pilot study regularly wore an activity sensor, which allowed their activity to be recorded objectively (see Figure 4). Such wearables have also been used in other studies to track physical activities [15,16]. The WHO guidelines [45] define whether a person gets enough exercise, which can be compared with the objectively measured amount of activity recorded using the activity sensor. On average, participants achieved the recommended 150 minutes of moderate activity per week (7 out of 10 participants did exceed this limit during the 4-week study period). The high SD also clearly shows that there are very large differences between individuals in terms of weekly activity (see Table 1). The observation that the achievement of the level of moderate activity recommended by the WHO can vary greatly from person to person is also reflected in the study by Jefferis et al [58]. In this cohort study, accelerometer data were collected using a GT3x accelerometer from a total of 2450 older adults aged between 70 and 93 years, of whom only 25% (n=613) achieved the recommended 150 minutes of moderate activity per week. Nevertheless, the Axivity AX6 proved to be a reliable wearable in this pilot study as in the study by Powell et al [59].

### Data Evaluation

Data on nutrition and thus data collection using the MNA-SF and a tablet-based food diary could also be recorded and evaluated. As a validated screening tool, the MNA-SF is very well suited for testing older adults for the risk of malnutrition [33]. With regard to the food diary, the EOS clearly showed that using the tablet app and entering data into the digital food diary caused the most difficulties (see Table 2). The participants stated that the tablet app often crashed or took a very long time when entering food into the food diary, and that some foods could not be found or were not sufficiently specified. In addition to the technical challenges that still need to be improved to make the tablet app run smoothly, the use of such apps presented challenges for older adults. Using the app can be difficult simply due to limitations in physiological functions, which make tapping buttons or scrolling difficult. In addition, the cognitive load can be very high for older adults, as they have to keep several navigation steps in mind to make entries in the diary [12,13].

In previous iterative studies, the app design was already adapted to the needs of older adults using a user-centered design [8,12,22,53]. In this study, care was taken to provide detailed instructions on how to use the tablet in order to reduce cognitive load and provide participants with the best possible support, as many older adults do not have much experience using apps on mobile devices [21,22]. The review by Zager Kocjan et al [19] also showed that it is important to closely involve older adults in the development of the assistance system or app to counteract the lack of trust in technology. In this study, participants were therefore closely supervised and were given printed instructions for the tablet app and for entering data in the food diary to take home with them. Nevertheless, it seemed that the technical component of the tablet app was the biggest problem in the pilot study, not the execution of the entries themselves. This must also be examined in the subsequent RCT study once the tablet app has been developed further, so that there are no more crashes or delays in the entries, as food diaries also offer older adults the potential to sharpen their focus on healthy and possibly better nutrition [13].

The comparison between 2 groups, one of which uses the AS-Tra system over a longer period of 12 weeks and the other which does not, will be recorded in a planned RCT study. This study started in May 2025 and is scheduled to run for 2 years. These results will allow a more detailed examination of the effect of the complex intervention used in this study.

Nevertheless, the successful completion of the pilot study suggests that the system is likely to have a positive impact on optimizing the mobility and nutritional status of older adults. Therefore, the AS-Tra system appears to be very promising in terms of preventive and independent measures for healthy aging in older adults.

### Limitations

Certain limitations of this study must be taken into account. First, selection bias cannot be ruled out as it must be assumed that participants who register for the study already have a general interest in good mobility and healthy nutrition. This also applies if older adults must have a deficit in mobility and/or nutrition to be included in the study.

Second, the assessment of the exclusion criterion regarding whether participants were able to understand the study content is based on the subjective assessment of the study team. However, this assessment is supported by the objective assessment of health status using the questionnaires and tests (MNA-SF and SPPB).

Third, it should be noted that, compared to measuring mobility using automated assessments and mobile devices, assessing nutritional status is fundamentally a subjective process, as it relies solely on self-reported information using tools such as food diaries [13,60]. Although methods such as image analysis tools, photo-based diaries, and chemical sensors are increasingly available for assessing nutrition, their application in everyday life is complex and subject to limitations in terms of feasibility, data protection, and ethical concerns [13,61]. The MNA is therefore currently considered a guideline and is recommended as a survey method despite its limitations due to subjectivity. That is why researchers rely on nonautomated, subjective information, which may be distorted by memory gaps or misreporting of, for example, portion sizes [18].

Fourth, the study was neither designed nor powered to prove the positive trends observed in health status. Therefore, they should be interpreted with extreme caution and are not generalizable to the general population.

Finally, the data were collected by the members of the study team who were themselves involved in the development of the AS-Tra system, which means that the same level of objectivity cannot be guaranteed as if the study had been conducted by an uninvolved person.

Future studies could focus on the objective collection of dietary data to prevent the tablet app from crashing frequently when using the food diary. In addition, the subsequent RCT can be used to examine the effectiveness of the AS-Tra system more closely, which could provide insightful findings on healthy aging in older adults.

### Conclusion

The main objectives of this pilot study were to test the usability and feasibility of collecting behavioral and health data using an MuTS, along with the associated app, in older adults (≥70 y) with deficits in mobility and/or nutrition over a period of 4 weeks in order to gain insights for a subsequent randomized controlled study.

Overall, the usability of the system was rated as good (SUS, mean 79.3, SD 13.4). However, the varying support needs of older people and the importance of personal interaction can influence the acceptance of the technical assistance system. Compared to simpler app-based systems, AS-Tra has a broader focus on mobility and nutrition and therefore has high requirements on design and user-friendliness, as well as active training opportunities and regular assessments to ensure that older adults can use it independently.

Data collection proved feasible using questionnaires, activity sensors, and automated assessments. The tablet-based food diary caused most of the difficulties due to technical problems (in 57% [17/30] of cases where participants wanted to make entries, crashes occurred). Despite these obstacles, participants rated the system and the overall assessment of its use positively. Improvements to the tablet app, particularly the food diary, will be essential for the RCT, especially since the report on the nutritional status is based mainly on subjective self-reports compared to automated mobility measurements.

The evaluation of potential health benefits from using the AS-Tra system will be investigated in a 12-week RCT, which started in May 2025.

## Supplementary material

10.2196/89681 Multimedia Appendix 1 Evaluation Overall System questionnaire and experience report.

## Abbreviations

5-CRT: 5-Time Chair Rise Test
AS-Tra: Assistance System for sustainable improvement of nutritional and mobility status of older people (≥70 y) under consideration of the transtheoretical model of health behavior change
aTUG: automatic Timed "Up and Go"
EOS: Evaluation Overall System
ER: Experience Report
MNA-SF: Mini Nutritional Assessment—Short Form
MuTS: measurement and training station
REDCap: Research Electronic Data Capture
SPPB: short physical performance battery
SUS: System Usability Scale
TTM: transtheoretical model of behavior change
TUG: Timed "Up and Go"
WHO: World Health Organization
