# Supplementary material for: A Digital Assistive System for Maintaining Nutrition and Mobility in Older Adults: Usability and Feasibility Findings From a Pilot Study
Source: JMIR Form Res. 2026 May 13;10:e89681. doi: 10.2196/89681 (PMC13170999; doi:10.2196/89681)
Supplement: Multimedia Appendix 1 [file formative-v10-e89681-s001.docx]

Appendix

| Participant ID:_________ | Evaluation Overall System | Measurement point: | 🞏 T2 |
| --- | --- | --- | --- |

| **In the following, we would like to focus on your experiences using the measurement and training station and the app. There are no right or wrong answers — we are interested in your personal experiences.**  For each question, you will find a line with numbers and at each end two opposite word pairs (for example, 'interesting' and 'uninteresting').  **Please mark the number on the scale from 1 to 5, that reflects your opinion the best.** If you do not wish to answer a question, select "no answer." | | | | | | | | | | | | | | | |
| --- | --- | --- | --- | --- | --- | --- | --- | --- | --- | --- | --- | --- | --- | --- | --- |
| **Question** | **Classification** | | | | | | | | | | | | | | **No Answer** |
| 1. The contents of the program were… |  | | | | | | | | | | | | | |  |
|  |  | ① | | ② | | | ③ | | ④ | | | ⑤ | |  |  |
|  | interesting | | | | |  | | | | | uninteresting | | | |  |
| 1. Using the devices in the measurement and training station was… |  | | | | | | | | | | | | | |  |
|  |  | | ① | | ② | | | ③ | | ④ | | | ⑤ |  |  |
|  | easy | | | | |  | | | | | difficult | | | |  |
| 1. The training on the Senso was … |  | | | | | | | | | | | | | |  |
|  |  | | ① | | ② | | | ③ | | ④ | | | ⑤ |  |  |
|  | easy | | | | |  | | | | | difficult | | | |  |
| 1. The visit process in the measurement and training station was … |  | | | | | | | | | | | | | |  |
|  |  | | ① | | ② | | | ③ | | ④ | | | ⑤ |  |  |
|  | structured | | | | |  | | | | | chaotic | | | |  |
| 1. Entering my own data in the nutrition diary was… |  | | | | | | | | | | | | | |  |
|  |  | | ① | | ② | | | ③ | | ④ | | | ⑤ |  |  |
|  | good | | | | |  | | | | | bad | | | |  |
| 1. The information from the app was … for my everyday life |  | | | | | | | | | | | | | |  |
|  |  | | ① | | ② | | | ③ | | ④ | | | ⑤ |  |  |
|  | useful | | | | |  | | | | | useless | | | |  |
| 1. Using the app on the tablet regularly in my daily routine was … for me |  | | | | | | | | | | | | | |  |
|  |  | | ① | | ② | | | ③ | | ④ | | | ⑤ |  |  |
|  | easy | | | | |  | | | | | difficult | | | |  |
| 1. Visiting the measurement and training station regularly in my daily routine was … for me |  | | | | | | | | | | | | | |  |
|  |  | | ① | | ② | | | ③ | | ④ | | | ⑤ |  |  |
|  | easy | | | | |  | | | | | difficult | | | |  |
| 1. Finding specific content in the app when I search for it was … for me |  | | | | | | | | | | | | | |  |
|  |  | | ① | | ② | | | ③ | | ④ | | | ⑤ |  |  |
|  | easy | | | | |  | | | | | difficult | | | |  |
| 1. Using the overall system was … for me |  | | | | | | | | | | | | | |  |
|  |  | | ① | | ② | | | ③ | | ④ | | | ⑤ |  |  |
|  | good | | | | |  | | | | | bad | | | |  |

| Participant ID:_________ | Evaluation Overall System | Measurement point: | 🞏 T2 |
| --- | --- | --- | --- |

1. Were there any contents or elements in the measurement and training station or in the tablet app that you thought could be improved and/or that often bothered you?
   If so, what were they specifically?

__________________________________________________________________________________________________________________________________________________________________________________________________________________________________________________________________________________________________________________________________________________________________________________________________________________________________________________________________

1. From your perspective, how did the process of participating in the study work? Where did you encounter any problems?

____________________________________________________________________________________________________________________________________________________________________________________________________________________________________________________________________________________________________________

______________________________________________________________________________________________________________________________________________________

1. What did you like most about the measurement and training station or the tablet app? Were there any contents, devices, or elements that you found particularly good?
   If so, which ones specifically?

__________________________________________________________________________________________________________________________________________________________________________________________________________________________________________________________________________________________________________________________________________________________________________________________________________________________________________________________________

1. To what extent and why do you think the measurement and training station with the tablet app would be suitable for motivating you to change your nutrition and physical activity habits?

__________________________________________________________________________________________________________________________________________________________________________________________________________________________________________________________________________________________________________________________________________________________________________________________________________________________________________________________________

1. Is there anything else you would like to share with us?

__________________________________________________________________________________________________________________________________________________________________________________________________________________________________________________________________________________________________________________________________________________________________________________________________________________________________________________________________

| Participant ID:_________ | Experience Report | Measurement point: | ☐ T0 ☐ T1 ☐ T2 ☐ T3 |
| --- | --- | --- | --- |

**Tablet experiences**
Were there any problems using the app on the tablet?

☐ Yes
☐ No

If yes, what problems were there?

__________________________________________________________________________________________________________________________________________________________________________________________________________________________________________________________________________________________________________________________________________________________________________________________________________________________

**Axivity sensor experiences**

Was wearing the sensor uncomfortable, or did the sensor disturb you during certain movements or while sleeping?

☐ Yes
☐ No

Were there any problems using the Axivity sensor?

☐ Yes
☐ No

**Measuring and training station experiences**
Were there any problems using the measuring and training station?

☐ Yes
☐ No

If yes, which elements of the measuring and training station did you find difficult?

☐ Hand strength
☐ Heart rate monitor
☐ Scale
☐ Sit-to-stand and walking test
☐ Sit-to-stand and sit-down test
☐ Training on the Senso
☐ Updating the tablet
☐ Using the app on the screen

Please describe the problems/difficulties you had:

__________________________________________________________________________________________________________________________________________________________________________________________________________________________________________________________________________________________________________________________________________________________________________________________________________________________

What did you find particularly easy when using the measuring and training station?

__________________________________________________________________________________________________________________________________________________________________________________________________________________________________________________________________________________________________________________________________________________________________________________________________________________________
